# Characterizing Trends in Lung Cancer Mortality Attributable to Airborne Environmental Carcinogens

**DOI:** 10.3390/ijerph182413162

**Published:** 2021-12-14

**Authors:** Mitchell Veith, Drury McAlarney, Xiaonan Xue, Thomas E. Rohan, H. Dean Hosgood

**Affiliations:** Department of Epidemiology and Population Health, Albert Einstein College of Medicine, Bronx, NY 10461, USA; Drury.McAlarney@einsteinmed.org (D.M.); xiaonan.xue@einsteinmed.org (X.X.); thomas.rohan@einsteinmed.org (T.E.R.)

**Keywords:** lung cancer, global burden of disease, air pollution, HAP, tobacco

## Abstract

Tracheal, bronchus, and lung (TBL) cancer is the leading cause of cancer death globally, but trends in TBL mortality attributable to tobacco, ambient particulate matter pollution (APMP), and household air pollution (HAP) were unequally distributed within global population subgroups over the last three decades. We used data from the Global Burden of Disease 2019 study to quantify the impact of sex, time, sociodemographic development index (SDI), and age for each exposure from 1990–2019. During that interval, tobacco dominated the TBL cancer mortality landscape, with its minimum global age-adjusted death rate of 16.71 deaths/100,000 (95% Uncertainty Interval (UI): 15.27–18.13) outstripping maximums of 3.85 deaths/100,000 (UI: 2.82–4.83) and 2.54 deaths/100,000 (UI: 1.69–3.54) for APMP and HAP, respectively. In 2019, tobacco male TBL death rates exceeded female rates by a factor of 4.4:1. Ratios of 1.9:1 for APMP and 2.1:1 for HAP were seen. Our analysis indicates that both-sex middle SDI and female low, low-middle, and high-middle SDI populations are suffering increasing tobacco TBL burden. Efforts producing successful global reductions in HAP-associated TBL mortality should continue, with attention to low SDI female death rate increases. Finally, except for high SDI populations, global APMP-attributable TBL cancer burden is increasing and represents a major health concern.

## 1. Introduction

Lung cancer is the leading cause of cancer death globally [1], with tracheal, bronchus, and lung (TBL) cancers accounting for 3.61% (95% Uncertainty Interval (UI): 3.36–3.83%) of all global deaths in 2019, up from 2.28% (UI: 2.19–2.39%) in 1990 [2]. It is well known that risk factors, including air pollution and tobacco exposure, have contributed to this increase [3,4]. However, the increase has been unequally distributed within subgroups of the global population over time. Additionally, the composition of risk factor attributions for TBL cancers has changed within the last three decades, with some arguing that rising air pollution levels signal the emergence of a “new smoking,” while others argue that tobacco use unequivocally remains the far larger public health concern [5,6,7]. The influences of these exposures on TBL mortality by sex, age, and time have been less comprehensively elucidated at a multifactorial level. For example, the global sex disparity in TBL cancer mortality has been well established, with global male TBL cancer deaths outpacing those in females by a factor of greater than 2:1 in 2017 [8]. However, a more detailed analysis of data like those available in the Global Burden of Disease Study 2019 (GBD 2019) dataset is needed to understand which exposure scenarios are contributing to the disparity over time by sociodemographic development indices (SDIs) and by age group. To robustly characterize evolving trends in TBL mortality attributable to tobacco, ambient particulate matter pollution (APMP), and household air pollution (HAP), we quantified the impact of sex, time, SDI, and age group on TBL mortality in the GBD 2019 study to provide a more nuanced understanding of global population trends in preventable TBL cancer deaths.

Recent studies have shed light on TBL incidence, DALYs, and mortality trends in the GBD 2017 and GBD 2019 data [9,10,11,12]. Wang et al. and Deng et al. described the dynamic temporal trends in TBL cancer burden across SDI, sex, and age group in GBD 2017 data. An editorial by Zhang et al. further explored the broader global health and policy implications of TBL burden trends in similar subgroups using GBD 2017 data. Since these works were published, the new GBD 2019 study has provided two additional years of data estimates and broadly improved mediation analysis between tobacco-, APMP-, and HAP-exposure-attributable outcomes [2]. Safiri et al. took advantage of the updated methodology to comprehensively examine country level trends in the GBD 2019 data for TBL-attributable DALYs stratified by sex, age, and time. Their group noted top risk factors contributing to TBL DALYs, stratified by age and world region, but did not pursue exposure-specific analysis or additional levels of group stratification. As such, no publication to date has provided analysis of temporal mortality trends for individual exposures in the GBD 2019 data stratified by sex, SDI, and age. Our work delves deeper for each exposure, expanding on the aforementioned studies and identifying targetable populations where primary preventive measures may be implemented. Furthermore, we provide unique heat map visualizations of this data to enable readers to easily interact with 594 subgroup temporal trends available from our analysis.

## 2. Materials and Methods

Annual TBL cancer deaths and death rate estimates attributable to environmental exposures were extracted from the GBD 2019 dataset [13]. Death rate estimates attributable to each exposure were stratified by sex, level of socioeconomic development (SDI), age, and calendar year. Estimates were produced using methodology described in the GBD 2019 study Supplementary Appendix 1, Sections 2 and 4 [2]. For all estimates, 95% Uncertainty Intervals (UIs) were available. From TBL mortality attributable risks, we selected tobacco, APMP, and HAP exposures for analysis, excluding other risk factors, like Ambient Ozone Pollution and specific occupational risks, to limit the scope of our analysis to three of the top preventable exposures causing TBL mortality.

Specific considerations for each stratifying variable were as follows: (1) Time: TBL deaths and age-standardized death rate estimates were available for all years between 1990–2019; (2) SDI: The GBD 2019 authors created SDI as a country’s “composite indicator of socio-demographic development status” utilizing “total fertility rate in those under 25 years old, mean education for those 15 years or older, and lag-distributed income per capita” to give each country an index value from 0 to 100 [2]. These SDI values were stratified into quintiles representing low- (LSDI), low-middle- (LMSDI), middle- (MSDI), high-middle- (HMSDI), and high-index (HSDI) countries; and (3) Age: Age-specific mortality was available for 5-year age groups between 40–90 years. Mortality below age 40 and above age 90, which may suggest TBL mortality attributed to factors related to genetic predisposition or longevity, was excluded from our analysis (Supplemental Figure S1). In our analysis, statistically significant differences in subpopulation death rates between 1990 and 2019 were determined. To do this, we conducted two sample *t*-tests of the 95% confidence interval of the difference between a given subgroup’s 1990 and 2019 death rates, at a significance level of *p* < 0.05 (Table 1, statistically significant *p*-values marked with “*”). Statistically significant findings are termed “significant” in the Results section; non-significant findings are not preceded by a word modifier. Lastly, using Microsoft Excel software, we generated heat maps for each exposure using death rate stratification by sex, SDI, and age between 1990 and 2019 to highlight granular trends among subpopulations. Other visualizations were generated using the GBD Compare tool [14].

## 3. Results

### 3.1. Tobacco

**Figure 1:**
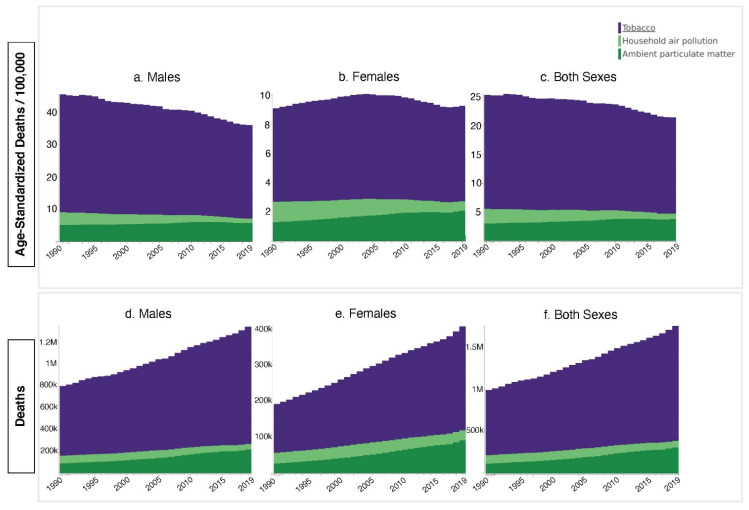
Global Tracheal, Bronchus, and Lung Cancer Mortality (Age-Standardized Deaths/100,000, Absolute Deaths) Attributed to Tobacco, Household Air Pollution, and Ambient Particulate Matter Pollution Exposures, by Sex, from 1990–2019.

From 1990–2019, tobacco dominated the TBL cancer mortality landscape. Tobacco-attributed global age-adjusted death rates ranged from 16.71 deaths/100,000 (UI: 15.27–18.13) to 19.99 deaths/100,000 (UI: 19.10–20.86), which outstripped interval maximum rates of 3.85 deaths/100,000 (UI: 2.82–4.83) and 2.54 deaths/100,000 (UI: 1.69–3.54) for APMP and HAP, respectively (Figure 1c). Global deaths and death rates for males surpassed those of females every year for all three exposures (Figure 1a,b,d,e). In 2019, tobacco-attributed male TBL death rates exceeded female by a factor of 4.4:1. Corresponding ratios of 1.9:1 for APMP and 2.1:1 for HAP were seen (data from Table 1).

**Figure 2:**
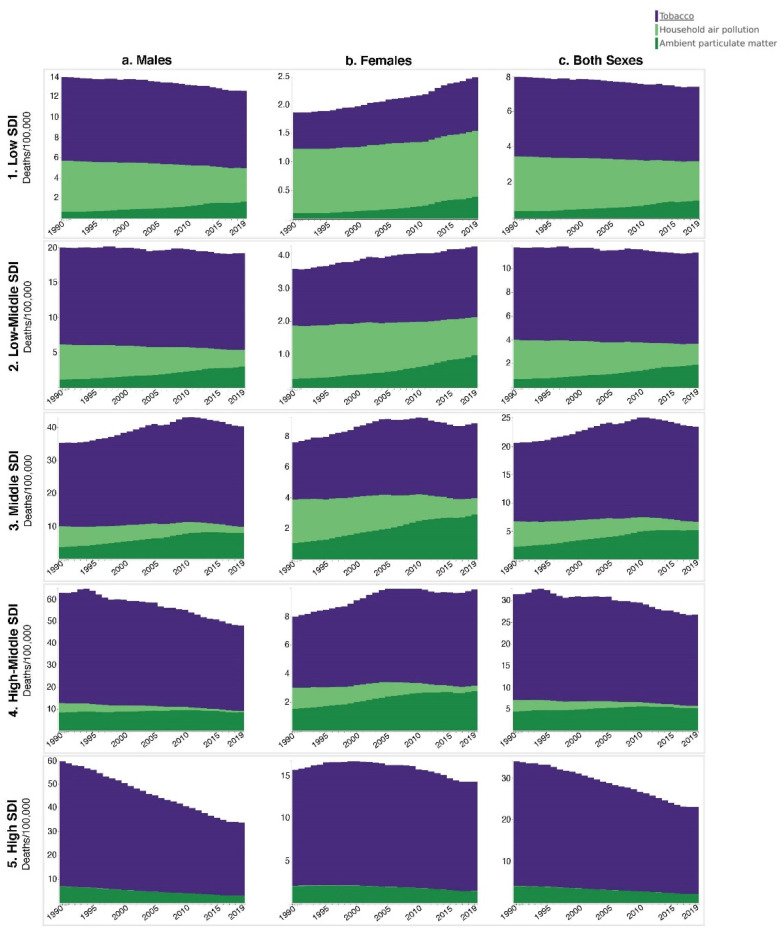
Global Tracheal, Bronchus, and Lung Cancer Mortality (Age-Standardized Deaths/100,000) Attributed to Tobacco, Household Air Pollution, and Ambient Particulate Matter Pollution by Sociodemographic Development Index (SDI) and Sex, from 1990–2019.

The global both-sex TBL tobacco mortality rate decreased significantly by 16% from 1990 to 2019 (*p* < 0.01). Males in the global population experienced a large, significant decrease (*p* < 0.01), while the female trend was a non-significant increase (*p* = 0.80). Exploring these trends by SDI highlighted significant decreases of 30% in HSDI (*p* < 0.01) and 14% in HMSDI (*p* = 0.01) both-sex populations (Figure 2(c4,c5)). Significant HSDI and HMSDI male decreases drove the overall decrease in both-sex populations, notably overshadowing a significant increase of 35% in the HMSDI female death rate (*p* < 0.01) (Figure 2(a4,a5,b4,b5)). Meanwhile, MSDI countries saw the only both-sex rate increase of 21% (*p* = 0.06) (Figure 2(c3)). An increasing trend was also observed for the separate results of male and female. MSDI males were the only male SDI age group that saw an increase (20%, *p* = 0.10); MSDI females saw a 31% increase (*p* = 0.07) (Figure 2(a3,b3,c3)). TBL mortality rates attributed to tobacco remained relatively unchanged between 1990–2019 for LSDI and LMSDI populations. Male mortality rates were 6.1-fold higher than female rates in these SDI strata; however, LSDI females saw a significant increase (*p* = 0.02) and LMSDI females a non-significant increase, while male subgroups saw non-significant decreases (Figure 2(a1,a2,b1,b2)).

### 3.2. APMP

Contrary to the global decrease in tobacco TBL mortality, global APMP death rates increased 25% from 3.03 deaths/100,000 (UI: 2.03–4.18) to 3.78 deaths/100,00 (UI: 2.79–4.86) between 1990–2019 (*p* = 0.35). The global male rate increased 12% to 5.78 deaths/100,000 (UI: 4.19–7.18), while the female rate increased 60% (*p* = 0.06) to 2.08 deaths/100,000 (UI: 1.49–2.71) (Figure 1a–c). HSDI countries, which had the highest APMP death rates in 1990, were the only SDI stratum that saw decreases by 2019. The both-sex HSDI population rate decreased 45% (*p* = 0.11), with similar decreases in the male and female rates during this period (Figure 2(a5,b5,c5)). Meanwhile, LSDI, LMSDI, and MSDI both-sex strata all saw > 100% APMP death rate increases, with significant increases of 171% for LMSDI (*p* = 0.01) and 133% (*p* < 0.01) for MSDI populations (Figure 2(c1–c3)). HMSDI both-sex populations saw an 18% increase (Figure 2(c4)). Notably, significant increases were present in all non-HSDI female substrata: 290% in LSDI (*p* = 0.01), 263% in LMSDI (*p* < 0.01), 178% in MSDI (*p* < 0.01), and 81% in HMSDI (*p* = 0.02). LMSDI and MSDI males additionally saw a significant increase of 157% (*p* = 0.01) and 119% (*p* = 0.01), respectively, while LSDI and LMSDI males also saw non-significant increases (Figure 2(a1–a3)). HMSDI males roughly maintained the 1990 APMP death rate of 8.43 deaths/100,000 (UI: 5.69–11.47) through 2019 (Figure 2(a4)).

### 3.3. HAP

While HAP continues to contribute to TBL mortality, the global HAP-attributable death rate saw a significant decrease from 1990–2019 of 62% (*p* = 0.01) from 2.54 deaths/100,000 (UI: 1.69–3.54) to 0.97 deaths/100,000 (UI: 0.55–1.93). The decrease in males was larger than that in females, with significant reductions of 66% (*p* = 0.01) in the former and a 54% reduction (*p* = 0.03) in the latter (Figure 1a–c). In 1990, HAP death rates exceeded those of APMP for LSDI, LMSD, and MSDI populations in both-sex, male, and female strata (Figure 2). The most notable difference at that time was present in LSDI both-sex populations: 3.09 deaths/100,000 (UI: 1.95–5.24) for HAP vs. 0.39 deaths/100,000 (UI: 0.12–0.86) for APMP. By 2019, unlike the general increasing trend of APMP death rates, HAP death rates fell in every SDI stratum for almost all both-sex, male, and female sub-populations. Of note, LMSDI female and LSDI both sex and male decreases were not significant, and LSDI females experienced a HAP death rate increase of 2.7% to 1.16 deaths/100,000 (UI: 0.08–1.64) by 2019 (Figure 2(b1)).

### 3.4. Age Group Analysis and Heat Maps

**Figure 3:**
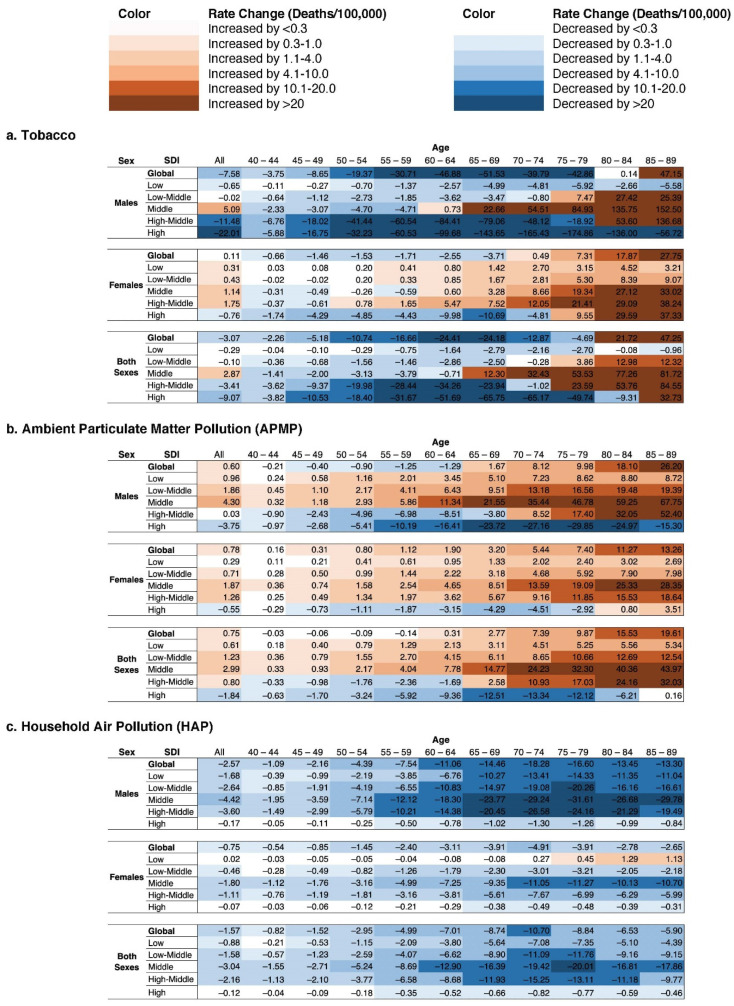
Changes in Tracheal, Bronchus, and Lung Cancer Mortality (Age-Standardized Deaths/100,000) Attributed to Tobacco, Ambient Particulate Matter Pollution, and Household Air Pollution Exposures, by Sex, Age Group, and Sociodemographic Development Index (SDI), from 1990–2019. Cell values represent the calculated difference between the 2019 and 1990 death rate for a given subgroup (2019 deaths/100,000–1990 deaths/100,000). Generated in Microsoft Excel.

The temporal trends in TBL mortality reported above also further varied within age groups. We were able to provide coherence to patterns in 594 five-year age groups between ages 40–90 for tobacco, APMP, and HAP exposures by creating heat maps (Figure 3), which indicate the magnitude of change between 1990 and 2019 in TBL death rates. The tobacco map (Figure 3a) revealed a skew toward large death rate increases in older age groups, while younger age groups generally saw decreases. Notable exceptions to this characterization included HSDI male populations, which saw large decreases across all age groups, and non-HSDI female populations, which saw death rate increases far more often in younger populations than their male counterparts.

The APMP map (Figure 3b) revealed a broad swath of TBL death rate increases, also skewing to larger increases in older age groups. The only age groups that escaped this trend were HMSDI males below age 70 and the full age range of HSDI both-sex, male, and female populations with the small exception of females above age 80. By contrast, the HAP map (Figure 3c) revealed near universal TBL mortality rate decreases between 1990 and 2019. Older LSDI females saw the lone rate increases within the HAP heatmap.

## 4. Discussion

Tracheal, bronchus, and lung cancer morbidity and mortality attributable to air pollution and tobacco have long been recognized as preventable. Understanding how specific exposures are contributing to morbidity and mortality patterns over time in subgroups of the global population is therefore essential to targeting preventive measures. As previously discussed, recent studies have described TBL burden in the GBD 2017 and GBD 2019 data, but either lack the use of the most recent GBD 2019 data or lack sufficiently robust analysis to identify statistically significant subpopulation trends for tobacco, APMP, or HAP exposures [9,10,11,12]. Much has remained consistent between the GBD 2017 and GBD 2019, including the methodology of modeling tobacco exposure-outcome pairs and broad trends in tobacco, APMP, and HAP-attributable TBL mortality. However, notable differences are present. As would be expected, the GBD 2019 data account for updated exposure data inputs in all three of our exposures of interest [2]. More importantly for our analysis, GBD 2019 methodology improved mediation analysis for APMP and HAP risk curves. This engenders greater confidence in the individual contributions of each of the three exposures on TBL burden, including TBL mortality. Few disparate trends existed between GBD 2017 and 2019 data although one example of an exception to this generalization occurred in in LSDI female populations. GBD 2017 data showed a significant decrease in LSDI female HAP-attributable TBL mortality, whereas GBD 2019 data did not in our analysis. With these distinctions in mind, our analysis was able to capitalize on the most up to date and comprehensive data available in GBD 2019 to identify exposure and mortality trends over the last three decades that have shaped the TBL mortality landscape.

The WHO reported in 2016 that tobacco smoke exposures have been trending downward in high-income countries while increasing in low- and middle-income countries [15]. This trend is borne out in our analysis, which found that tobacco-attributable TBL mortality rates decreased in HSDI populations but increased modestly in HMSDI and significantly in MSDI populations. Both-sex tobacco death rates remained relatively constant in LSDI and LMSDI populations, though this masked divergent male and female trends in these SDI strata. Historically, the global sex difference in TBL mortality was reliably attributed to sex-specific behavioral differences (e.g., male tobacco use at higher rates). It is now likely that behavioral changes in high-income countries, like the United States, have caused male and female TBL mortality rates to converge [16]. We found this generalization to be supported by our analysis. HSDI males experienced a sharp decline, while females saw a modest decline or even an increase in older age groups over the past 29 years. Likewise, younger HMSDI males saw rates decline while females in almost all corresponding age groups saw increases during the same period. Though LSDI, LMSDI, and MSDI male populations maintained higher mortality rates than their female counterparts, females saw increases across a greater percentage of age groups. These findings are reminiscent of historical tobacco-driven mortality trends in high-income countries, suggesting a concerning future trajectory for low-income countries.

As tobacco behaviors changed around the world in the last three decades, APMP levels have increased. In 2019, 92% of the world’s population was living an area that exceeds the WHO’s guideline for healthy air [17]. Conversely, HAP exposures have seen a significant global reduction despite continuing to plague rural populations in many low- and middle-income countries [17]. Emerging evidence also suggests that these exposures may be associated with a growing list of other health problems, including cardiorespiratory diseases, cancers of additional anatomical sites, and diabetes [18,19,20]. With these exposure-risk trends in mind, age-stratified analyses of APMP and HAP death rates may offer a signal for future TBL mortality trends as current global populations age.

In age groups < 65 years, increases in APMP-attributable TBL death rates over the last three decades for LSDI, LMSDI, and MSDI populations paint a concerning picture. High APMP death rates seen in almost every > 65 years age group already represent a major issue in the global TBL disease burden. If patterns in younger age groups portend a continuation of increasing APMP mortality rates as the population ages, this crisis will worsen. Countries like the U.S. (HSDI) and, more recently, China (HMSDI) do offer some indication that implementing regulations aimed at improving APMP levels can reduce related TBL mortality [5]. Our analysis found that almost all HSDI age groups saw decreases between 1990 and 2019, which may be attributable to the higher likelihood of strong air pollution control efforts in those countries. Similarly, HMSDI males aged < 65 years saw decreases, a result likely driven by recent aggressive air pollution reduction efforts in China, the most populous HMSDI country [21]. HMSDI female groups aged < 65 years, however, did experience increases in the APMP death rate, demonstrating that further study is needed for this critical subpopulation.

HAP trends offered the most universally positive outlook among the three exposures studied. HAP-attributable TBL mortality reductions across almost every global subpopulation can be taken as a sign that international efforts to reduce the burden of disease associated with these exposures, such as indoor stove interventions [22,23], have been fruitful. However, HAP mortality continues to rival that of APMP in LSDI and LMSDI populations. LSDI female populations that have not benefitted from the global TBL death rate decrease represent a subgroup where preventive intervention may be most helpful. Future efforts need to focus on improving the health of this vulnerable subpopulation.

## 5. Conclusions

Our findings further characterize the complex, dynamic, exposure-related trends in TBL cancer mortality from 1990–2019. Our analysis indicates that the most urgent areas for tobacco-related intervention are both-sex MSDI populations and female LSDI, LMSDI, and HMSDI populations suffering from increasing tobacco-attributable TBL burden. Successful efforts that have brought about global reductions in HAP-associated TBL burden should continue, with special attention to LSDI female populations, where death rate increases were seen. Finally, except for both-sex HSDI and younger male HMSDI populations, the TBL cancer burden attributed to APMP is increasing and represents an urgent threat to the health of many global populations. With these targeted insights, global leaders, policy makers, and health care professionals will be better able to address the contribution of TBL cancer to the global burden of disease.

## Figures and Tables

**Table 1:** Changes from 1990 to 2019 in Tracheal, Bronchus, and Lung Cancer Mortality (Age-Standardized Deaths/100,000) Attributed to Tobacco, Ambient Particulate Matter Pollution, and Household Air Pollution Exposures, by Sex, Age Group, and Sociodemographic Development Index (SDI). A “*” denotes statistical significance in the difference between 1990 and 2019 death rates.

Exposure	Sex	SDI	1990 Death Rate (UI)	2019 Death Rate (UI)	*p*-Value
Tobacco	Males	Global	36.42 (34.60–38.54)	28.84 (26.02–31.72)	<0.01 *
		LSDI	8.26 (6.43–10.16)	7.61 (6.40–9.00)	0.59
		LMSDI	13.81 (11.90–16.22)	13.8 (12.42–15.22)	0.99
		MSDI	25.52 (22.74–28.69)	30.61 (25.70–35.77)	0.10
		HMSDI	50.48 (47.61–53.71)	38.99 (34.39–43.55)	<0.01 *
		HSDI	52.66 (51.06–53.97)	30.66 (28.91–32.02)	<0.01 *
	Females	Global	6.45 (5.99–6.92)	6.56 (5.86–7.26)	0.80
		LSDI	0.63 (0.49–0.83)	0.94 (0.78–1.10)	0.02 *
		LMSDI	1.71 (1.45–2.01)	2.14 (1.80–2.55)	0.10
		MSDI	3.71 (3.08–4.41)	4.86 (3.94–5.86)	0.06
		HMSDI	4.97 (4.39–5.58)	6.72 (5.85–7.71)	<0.01 *
		HSDI	13.63 (12.94–14.21)	12.87 (11.84–13.67)	0.13
	Both Sexes	Global	19.78 (18.85–20.83)	16.71 (15.27–18.13)	<0.01 *
		LSDI	4.46 (3.52–5.45)	4.17 (3.55–4.86)	0.64
		LMSDI	7.74 (6.75–8.96)	7.64 (6.89–8.39)	0.89
		MSDI	13.97 (12.55–15.59)	16.84 (14.36–19.37)	0.06
		HMSDI	24.34 (23.07–25.84)	20.93 (18.80–23.01)	0.01 *
		HSDI	29.90 (28.93–30.68)	20.83 (19.53–21.78)	<0.01 *
APMP	Males	Global	5.18 (3.49–7.13)	5.78 (4.19–7.48)	0.65
		LSDI	0.68 (0.21–1.52)	1.65 (0.95–2.51)	0.11
		LMSDI	1.17 (0.50–2.13)	3.03 (1.99–4.08)	0.01 *
		MSDI	3.60 (2.01–5.51)	7.89 (5.53–10.42	0.01 *
		HMSDI	8.43 (5.69–11.47)	8.46 (6.11–11.04)	0.99
		HSDI	6.92 (4.04–10.45)	3.17 (2.11–4.52)	0.05
	Females	Global	1.30 (0.85–1.81)	2.08 (1.49–2.71)	0.06
		LSDI	0.10 (0.03–0.21)	0.39 (0.22–0.59)	0.01 *
		LMSDI	0.27 (0.13–0.46)	0.98 (0.62–1.37)	<0.01 *
		MSDI	1.05 (0.58–1.69)	2.92 (2.06–3.89)	<0.01 *
		HMSDI	1.54 (1.00–2.15)	2.79 (2.02–3.66)	0.02 *
		HSDI	1.98 (1.13–3.08)	1.43 (0.91–2.09)	0.40
	Both Sexes	Global	3.03 (2.03–4.18)	3.78 (2.79–4.86)	0.35
		LSDI	0.39 (0.12–0.86)	1.00 (0.59–1.50)	0.08
		LMSDI	0.72 (0.32–1.28)	1.95 (1.28–2.63)	0.01 *
		MSDI	2.25 (1.26–3.44)	5.24 (3.79–6.77)	<0.01 *
		HMSDI	4.48 (3.01–6.11)	5.28 (3.90–6.80)	0.48
		HSDI	4.05 (2.33–6.12)	2.21 (1.45–3.16)	0.11
HAP	Males	Global	3.91 (2.54–5.59)	1.34 (0.72–2.15)	0.01 *
		LSDI	5.02 (3.09–8.53)	3.33 (2.13–4.84)	0.39
		LMSDI	5.01 (3.32–7.24)	2.37 (1.37–3.50)	0.04 *
		MSDI	6.33 (4.05–7.24)	1.91 (0.86–3.41)	<0.01 *
		HMSDI	4.31 (2.49–6.56)	0.72 (0.26–1.53)	<0.01 *
		HSDI	0.19 (0.08–0.35)	0.02 (0.00–0.05)	0.04 *
	Females	Global	1.40 (0.96–1.97)	0.65 (0.37–1.02)	0.03 *
		LSDI	1.13 (0.71–1.97)	1.16 (0.80–1.64)	0.95
		LMSDI	1.61 (1.07–2.36)	1.15 (0.73–1.63)	0.31
		MSDI	2.84 (1.92–3.90)	1.05 (0.52–1.75)	0.01 *
		HMSDI	1.49 (0.95–2.10)	0.39 (0.16–0.75)	<0.01 *
		HSDI	0.08 (0.04–0.14)	0.01 (0.00–0.03)	0.03 *
	Both Sexes	Global	2.54 (1.69–3.54)	0.97 (0.55–1.53)	0.01 *
		LSDI	3.09 (1.95–5.24)	2.21 (1.47–3.11)	0.46
		LMSDI	3.31 (2.26–4.70)	1.73 (1.05–2.47)	0.05 *
		MSDI	4.49 (2.99–6.20)	1.45 (0.71–2.47)	<0.01 *
		HMSDI	2.69 (1.62–3.93)	0.53 (0.21–1.09)	<0.01 *
		HSDI	0.13 (0.06–0.23)	0.01 (0.00–0.04)	0.02 *

## Data Availability

The data that support the findings of this study are openly available on the IHME GHDx web page at http://ghdx.healthdata.org/gbd-2019, reference number [13]. Accessed on 15 November 2020.

## Supplementary Materials

The following are available online at https://www.mdpi.com/article/10.3390/ijerph182413162/s1, Figure S1: Deaths by Exposure and Age Group in 2019 for Tracheal, Bronchus, and Lung Cancer Mortality.
