# Supplementary material for: Characterizing Trends in Lung Cancer Mortality Attributable to Airborne Environmental Carcinogens
Source: Int J Environ Res Public Health. 2021 Dec 14;18(24):13162. doi: 10.3390/ijerph182413162 (PMC8701182; doi:10.3390/ijerph182413162)
Supplement: Supplementary file 1 [file ijerph-18-13162-s001.zip › ijerph-1450941-supplementary.pdf]

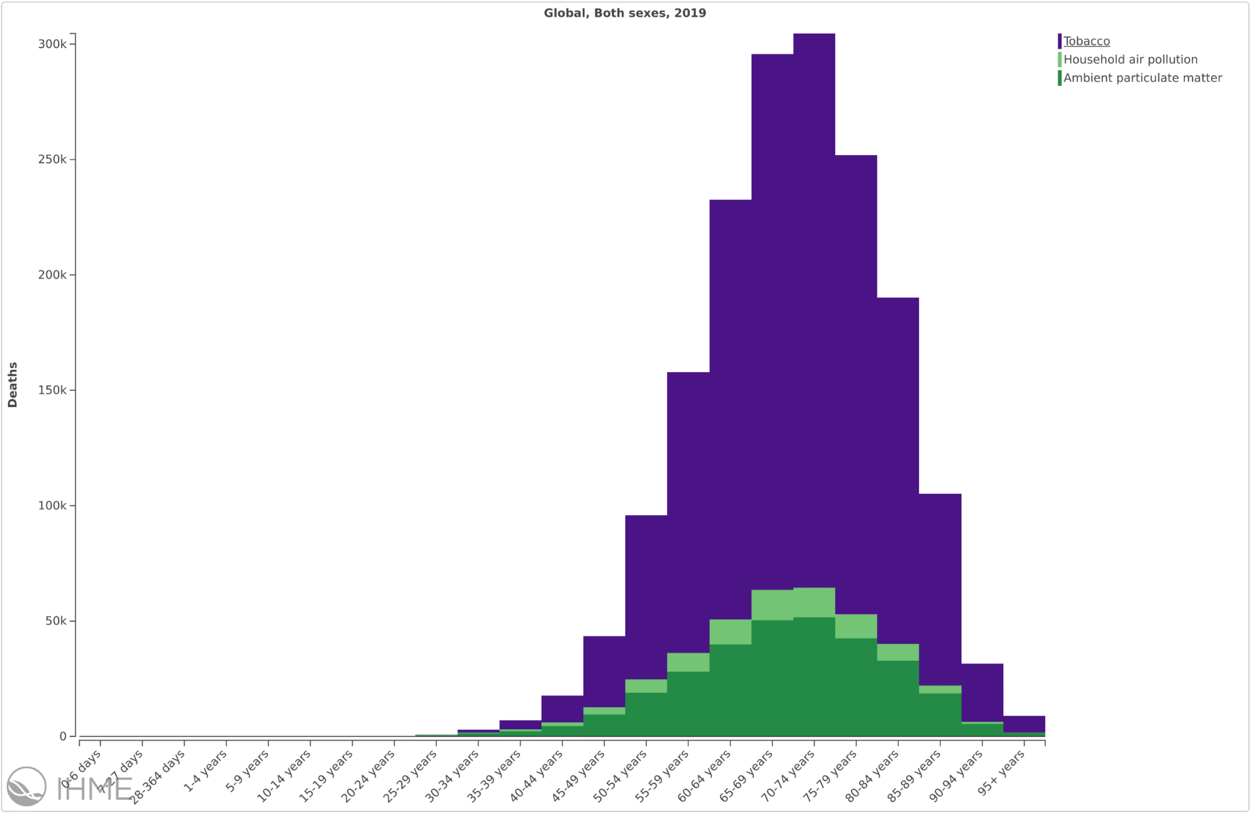


**Supplemental Figure S1:** Global Tracheal, Bronchus, and Lung Cancer Deaths by Attributable Exposure and Age Group in 2019
